# The Impact of Posterior Occlusal Support Recovery by Dental Implant Treatment on the Survival of Remaining Teeth: A Long-Term Retrospective Study

**DOI:** 10.3390/jcm14248939

**Published:** 2025-12-18

**Authors:** Fumiko Nakamura, Yohei Jinno, Yo Yamasaki, Michiko Furuta, Toru Takeshita, Yasushige Sakamoto, Takatsuna Nakamura, Yasunori Ayukawa

**Affiliations:** 1 Section of Implant and Rehabilitative Dentistry, Division of Oral Rehabilitation, Faculty of Dental Science, Kyushu University, Fukuoka 812-8582, Japan; fumiko.nakamura@dent.kyushu-u.ac.jp (F.N.); sakamoto-y@dent.kyushu-u.ac.jp (Y.S.); ayukawa@dent.kyushu-u.ac.jp (Y.A.); 2 Nakamura Dental Clinic, Kumamoto 863-0031, Japan; yamayo1984@gmail.com (Y.Y.); nakamura.t@proof.ocn.ne.jp (T.N.); 3 Section of Preventive and Public Health Dentistry, Division of Oral Health, Growth and Development, Faculty of Dental Science, Kyushu University, Fukuoka 812-8582, Japan; mfuruta@dent.kyushu-u.ac.jp (M.F.); taketooo@dent.kyushu-u.ac.jp (T.T.); 4 Center for Dental Implants KYUSHU Dental Clinic, Kumamoto 860-0802, Japan

**Keywords:** dental implant, occlusal support, Eichner classification, retrospective cohort study

## Abstract

**Background**: Preservation of posterior occlusal support is critical for maintaining residual dentition in partially edentulous patients. Although implant-supported restorations are widely used, long-term evidence on their role in preventing tooth loss remains limited. **Methods**: A retrospective cohort study of 225 patients (80 males, 145 females; mean age 53 ± 9 years) who received posterior implant-supported restorations, with a mean follow-up of 21 years. Post-treatment occlusal support was classified using the Eichner classification. Tooth and implant loss were recorded. Associations between residual occlusal support and tooth loss were analyzed using Pearson’s chi-square test, with thresholds of ≥1, ≥2, or ≥3 teeth lost. Multivariate logistic regression analyses adjusted for age and gender were performed to evaluate the independent association of the Eichner classification. **Results**: After prosthesis delivery, 159 patients were classified as Eichner A, 48 as B1, 16 as B2, and 2 as B3. The mean number of lost teeth was 2.4, and 35 implants failed in 22 patients. Tooth loss ≥3 occurred more frequently in the Eichner B groups than in A. (48.5% vs. 34.0%). In multivariate analyses, Eichner classification significantly predicted loss of ≥3 teeth (adjusted OR = 1.83; 95% CI: 1.00–3.33; *p* = 0.048), but not ≥1 or ≥2 teeth. **Conclusions**: Posterior implant therapy provides durable occlusal support and contributes to long-term preservation of residual dentition. Patients with limited occlusal support remain at a higher risk of substantial tooth loss, even after adjustment for age and gender. The Eichner classification represents a practical clinical tool for evaluating occlusal support and predicting long-term prognosis in partially edentulous patients.

## 1. Introduction

The prosthodontic management of partial edentulism has undergone a paradigm shift with the widespread integration of dental implant therapy into contemporary treatment protocols. While traditional modalities—including fixed dental prostheses (FDPs) and removable partial dentures (RPDs)—have long been the standard of care, their biomechanical limitations are well-documented. FDPs require irreversible preparation of adjacent teeth, whereas RPDs can subject abutment teeth to deleterious biomechanical loading, thereby accelerating periodontal breakdown and abutment tooth loss [1,2].

Such restorative compromises may precipitate a cascade of unfavorable outcomes, notably the expansion of edentulous spans and progressive occlusal deterioration, ultimately culminating in the collapse of the occlusal scheme [3]. The concept of occlusal collapse encompasses a multifactorial decline in functional occlusal support, often characterized by loss of vertical dimension, compromised mastication, and increased prosthodontic complexity [4,5,6,7]. Implant-supported restorations, by re-establishing posterior occlusal support independent of adjacent dentition, offer a biologically and biomechanically favorable alternative. However, there is a paucity of long-term evidence elucidating the extent to which implant-mediated occlusal support contributes to the maintenance of occlusal integrity and the prevention of progressive collapse.

**Figure 1:**
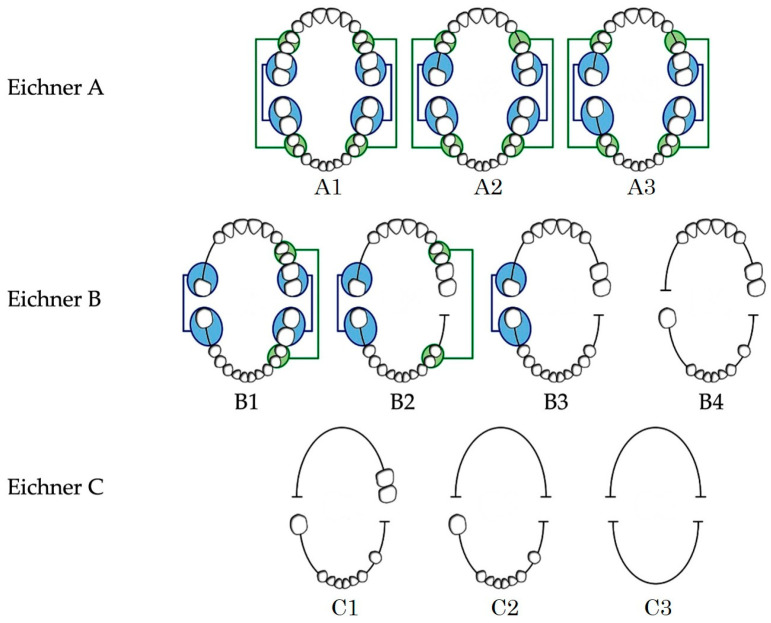
Eichner classification. Blue or green indicates the presence of posterior occlusal support.

The Eichner classification divides occlusal support into three primary categories based on the presence or absence of occlusal contacts in four supporting zones, located in the premolar and molar regions on both sides. Class A represents full support in all zones. Class B indicates partial or complete loss of posterior support, provided that anterior occlusal contacts are preserved. Class C signifies no occlusal contacts in both the posterior and anterior teeth (see Figure 1) [8]. Since inadequate posterior support increases functional loading on remaining teeth, the Eichner classification is regarded as a clinically meaningful indicator of biomechanical stability. For these reasons, we chose the Eichner classification as the primary outcome in this study. According to a previous report using a large population-based cohort study, decreased posterior occlusal support, as classified by the Eichner classification, was a risk indicator for tooth loss. This tendency was emphasized in anterior tooth loss [3]. These results may indicate the usefulness of the Eichner classification for predicting the prognosis of natural teeth, and it can be anticipated that this classification is also suitable for predicting the prognosis of dental implants.

Conventional prosthodontic strategies, such as cross-arch splinting via FDPs or telescopic crown-retained RPDs, have been advocated to provide rigid stabilization of the arch. Although these approaches may confer short- to medium-term benefits in load distribution and occlusal stability, their long-term efficacy is limited by biological and mechanical complications, including abutment tooth fracture, endodontic compromise, and hygiene-related periodontal deterioration [9,10]. Accordingly, the long-term effectiveness of such approaches in halting or slowing occlusal collapse remains questionable.

In contrast, posterior implant placement allows for the direct reconstitution of functional occlusal support without further compromising the natural dentition. Recent studies utilizing occlusal diagnostic tools, such as pressure-sensitive occlusal films (e.g., Dental Prescale^®^), have demonstrated that posterior implants effectively redistribute occlusal forces, alleviating excessive anterior loading and reducing stress on teeth adjacent to edentulous areas [11,12]. These biomechanical improvements may translate into prolonged preservation of residual teeth and enhanced occlusal stability.

Nevertheless, most investigations into the occlusal benefits of posterior implants have been limited to short-term observation periods. To date, there remains a significant gap in the literature regarding the long-term impact of implant-mediated posterior occlusal support on the prevention of occlusal collapse and the preservation of the residual dentition.

The present study aims to address this knowledge gap by retrospectively evaluating patients who received posterior implant-supported restorations and were followed for over 15 years. Specifically, we seek to elucidate the relationship between long-term restoration of posterior occlusal support and subsequent changes in occlusal support classification, residual dentition, and overall prosthodontic stability.

## 2. Materials and Methods

This study was approved by the Clinical Research Ethics Committee of the Japanese Society of Oral Implantology (Approval Number: 2025-9-11000694).

### 2.1. Study Design and Population

A retrospective cohort study was conducted among patients who underwent implant therapy at Nakamura Dental Clinic and the Center for Dental Implants KYUSHU Dental Clinic between 1 January 1985 and 31 December 2009, with a minimum follow-up of more than 15 years after final prosthesis placement. Thus, this study reflects a long-term maintenance cohort rather than an inception cohort of all implant-treated patients. Since patients who discontinued maintenance many years ago no longer had complete or retrievable clinical records, the exact number of initially treated patients could not be accurately determined. Inclusion/exclusion criteria are as follows:

#### 2.1.1. Inclusion Criteria

- Male or female patients aged 20 years or older at the time of implant placement
- Patients who received implant therapy specifically in the posterior dentition (premolar and molar regions)
- Patients with documented clinical and radiographic follow-up data extending beyond fifteen years after definitive prosthetic rehabilitation

#### 2.1.2. Exclusion Criteria

- Patients with uncontrolled systemic diseases that could affect oral or implant health
- Patients presenting with active periodontal disease at the baseline assessment
- Patients whose follow-up duration was insufficient for long-term evaluation
- Patients with incomplete or inadequate clinical documentation
- Patients unable to attend regular maintenance visits

### 2.2. Data Extraction

The following data were extracted from the patients’ clinical records and radiographic examinations:

1. Patient demographics and treatment-related information, including age, sex, date of implant placement, and date of superstructure delivery.
2. The occlusal support area at the time of final superstructure delivery, classified according to the Eichner classification.
3. The condition of the missing teeth, including the presence or absence of endodontic treatment and restorative treatment, the positional relationship with implant-supported superstructure (antagonistic and mesiodistal relationships), the reason for tooth extraction, and the date of extraction.
4. Information regarding failed implants, including the implant site and the date of implant loss.

### 2.3. Statistical Analysis

The association between the number of lost teeth and the Eichner classification (A group vs. B group) was initially assessed using Pearson’s chi-square test. To examine whether the degree of tooth loss differed by occlusal support status, three threshold levels were examined (≥1, ≥2, or ≥ 3 teeth lost). For each threshold, a 2 × 2 contingency table was constructed and analyzed. Statistical significance was set at *p* < 0.05.

Multivariate logistic regression analyses were performed to adjust for two potential confounding factors: age and gender. For each threshold level of tooth loss, logistic regression models were constructed using age and gender, and Eichner classification as explanatory variables. Adjusted odds ratios (ORs) and 95% confidence intervals (CIs) were calculated to evaluate the independent association between occlusal support and tooth loss. All patients had at least 15 years of follow-up after receiving the definitive prostheses. Although all participants were observed for an extended period, the exact duration of follow-up was not reported, which could introduce bias.

To complement the primary analyses, we additionally estimated risk ratios and risk differences using binomial regression models and assessed model discrimination using the C-statistic derived from the predicted probabilities of the logistic regression models. We also tested negative binomial models in which tooth-loss counts were treated as continuous. Detailed results of these supplemental analyses are presented in the Supplementary Materials.

The thresholds for tooth loss (≥1, ≥2, and ≥ 3 teeth) were selected because the distribution of tooth loss in this cohort was highly skewed, with most patients losing one to three teeth and only three individuals losing ten teeth or more. Minor tooth loss (one or two teeth) is commonly attributable to non-occlusal causes such as endodontic failure, vertical root fracture, or localized caries. In contrast, the loss of three or more teeth is more likely to reflect cumulative biomechanical loading. Therefore, these clinically meaningful thresholds were adopted.

Only age and sex were included as covariates because major confounding factors, including smoking status, periodontal condition, systemic diseases, oral-hygiene level, and parafunctional habits, were not consistently documented in the long-term clinical records.

All statistical procedures were performed using Stata SE version 18.0 (Stata Corp LP, College Station, TX, USA).

## 3. Results

### 3.1. Patient and Implant/Prosthesis Outcomes

A total of 225 patients were included in the analysis (80 males and 145 females) (Table 1). The mean age at baseline was 53.04 ± 9.22 years old. The mean follow-up period was 255.55 ± 56.32 months. The mean number of remaining teeth before the treatment was 21.65 ± 3.85. The loss rate of dental implants was 5.0% and the average service time of definitive prosthesis was 21.4 ± 4.5 years.

### 3.2. Occlusal Support Status After Treatment

Eichner classifications after the delivery of definitive prostheses were as follows: Eichner A: 159 patients (70.67%), B1: 48 patients (21.33%), B2: 16 patients (7.11%), and B3: 2 patients (0.89%) (Table 2).

### 3.3. Tooth Loss During the Observation Period

The mean number of lost teeth in each patient during the follow-up period was 2.43 ± 2.45. The distribution of tooth loss was as follows: 0 teeth (*n* = 55), 1 (*n* = 50), 2 (*n* = 34), 3 (*n* = 22), 4 (*n* = 21), 5 (*n* = 19), 6 (*n* = 5), 7 (*n* = 11), 8 (*n* = 2), 9 (*n* = 3), 10 (*n* = 1), 11 (*n* = 1), and 13 (*n* = 1). The total number of lost teeth was 547 (Table 3). The distribution of tooth-loss counts ranged from 0 to 13 teeth. Although the variability was wide, only three patients lost 10 or more teeth, and these extreme cases did not materially affect the overall statistical associations.

### 3.4. Implant Loss

Regarding implant failures, 203 patients experienced no implant loss, whereas 15 patients lost one implant, three patients lost two implants, three patients lost three implants, and one patient lost five implants. In total, 35 implants were lost during the observation period (Table 4).

### 3.5. Association Between Tooth Loss and Eichner Classification After the Definitive Prosthesis Delivery

To clarify the relationship between occlusal stop and tooth loss, three predefined thresholds were analyzed: ≥1, ≥2, and ≥3 lost teeth.

For each threshold, a cross-tabulation table comparing the Eichner A and Eichner B groups was created, and Pearson’s chi-square test was conducted. No significant difference was found between the groups for ≥1 lost tooth (*p* = 0.080) or ≥2 lost teeth (*p* = 0.159).

However, patients classified as Eichner B had a significantly higher frequency of losing ≥3 teeth than those in Eichner A (*p* = 0.041). These results suggest that the impact of impaired posterior occlusal support becomes apparent only when tooth loss reaches a more severe level (Table 5).

### 3.6. Multivariable Logistic Regression Adjusted for Age and Gender

After the adjustment for age and gender, Eichner classification was not significantly associated with loss of ≥1 tooth (adjusted OR = 1.88; 95% CI: 0.89–4.00; *p* = 0.099) or loss of ≥2 teeth (adjusted OR = 1.37; 95% CI: 0.74–2.51; *p* = 0.317). In contrast, when the threshold was defined as ≥3 lost teeth, patients classified as Eichner B had a statistically significantly higher risk of tooth loss than those classified as Eichner A (adjusted OR = 1.83; 95% CI: 1.00–3.33; *p* = 0.048). These results indicate that a significant association with occlusal support is present only in cases involving more severe levels of tooth loss (Table 6).

In summary, among the subjects with three or more missing teeth, the tooth loss was significantly correlated with Eichner classification: a higher chance of tooth loss in the Eichner B group than in the Eichner A group.

## 4. Discussion

The cumulative survival rate of implants in this study was approximately 95%. This is consistent with previous research findings [13].

This study examined the impact of the Eichner classification following implant treatment on the prognosis of remaining natural teeth. The association was further evaluated using multivariable logistic regression, adjusting for age and sex, to assess the independent effects of each factor on tooth loss. The results suggest that among patients in the group who lost three or more natural teeth during the observation period, those with Eichner classification B after delivery of the definitive prosthesis had a significantly higher risk of residual tooth loss than those with classification A. This may be attributed to increased functional load on the remaining teeth. Indeed, previous studies have reported that, without a posterior prosthesis, occlusal forces on the remaining anterior teeth increase [14].

Furthermore, even in patients with relatively good occlusal conditions, a certain degree of tooth loss was observed over time, emphasizing the importance of continuous monitoring and preventive management [15]. These findings support the necessity of individualized treatment plans, appropriate distribution of occlusal load, and preventive strategies for the long-term preservation of the remaining dentition.

The primary reason for an Eichner classification of B after treatment is often financial constraints. Additionally, anatomical constraints sometimes prevent posterior implant placement. However, this study suggested that cases that do not achieve Eichner Class A after implant treatment—meaning incomplete restoration of molar occlusal support—pose a risk to the prognosis of remaining natural teeth. This highlights the importance of fully restoring occlusal support.

Conversely, when subjects who lost one or two natural teeth during the observation period were included, this trend disappeared. This likely indicates that factors other than the Eichner classification influence tooth survival. One possible explanation for this pattern is that non-occlusal factors, including endodontic failure, vertical root fracture, or localized caries, often lead to the loss of one or two teeth. These findings suggest that the association between incomplete posterior occlusal support and the loss of three or more teeth may be influenced by increased functional loading on the remaining dentition; however, this interpretation should be considered hypothetical, as biomechanical factors were not evaluated in the present study. Finite element analyses have demonstrated that insufficient posterior occlusal support can lead to concentrated mechanical stress on implant-supported prostheses and surrounding bone, particularly under oblique loading, offering a plausible biomechanical context for the trends observed in this study [16]. Therefore, the relationship identified here should be interpreted as an association rather than a causal effect. When posterior support cannot be fully restored due to anatomical or financial limitations, closer exceptional follow-up and individualized management may help reduce potential functional loading on both implants and remaining teeth.

Several limitations of this pilot study should be acknowledged. The retrospective study design limits causal inferences and introduces potential selection bias. Furthermore, the relatively small sample size of patients with limited occlusal support limits the generalizability of subgroup analyses. In particular, the minimal number of patients classified as Eichner B2 or B3 substantially limited the statistical power of subgroup analyses. This may have attenuated or obscured potential differences between subclasses within the B group. Additionally, factors such as systemic conditions, smoking, abnormal occlusal habits, and oral hygiene were not evaluated, yet they could influence the survival rates of both natural teeth and implants. Furthermore, although the nature of the opposing dentition (implant or natural tooth) is thought to significantly impact the prognosis of the remaining natural teeth, as previously reported [17], this aspect was not analyzed in the present study. Another limitation is the absence of several important occlusal and prosthetic variables. Information regarding opposing dentition conditions, parafunctional habits such as bruxism, periodontal history, occlusal schema, prosthodontic design, and implant distribution was not consistently documented in the long-term clinical records. Because these factors may influence occlusal loading and tooth-survival outcomes, their absence limits the interpretability of the present findings. This study analyzed only pure titanium implants and excluded short implants (less than 6 mm). However, factors such as implant material, length, and diameter may influence the results. Short implants have reportedly shown no difference in survival rate compared with conventional implants [18,19]. Moreover, although Poisson and negative binomial models were explored to model tooth loss as a count variable, stable parameter estimation was not feasible due to the highly skewed distribution and small subgroup sizes.

Other limitations include variation in follow-up time among patients. Although all participants were followed for more than 15 years, differences in the exact length of observation may have affected the rates of tooth loss or implant failure. Future prospective studies that include time-to-event analyses are necessary. Additionally, the specific causes of tooth loss were not documented in this study. Non-occlusal factors, such as endodontic failure, vertical root fractures, or localized caries, may have contributed to tooth loss, thereby weakening the observed link between occlusal support and the Eichner classification. Moreover, the study population consisted exclusively of long-term maintenance attendees, which inevitably introduces selection bias. Important potential confounders, including smoking status, systemic diseases, periodontal condition, oral hygiene level, and bruxism, were not recorded in the available clinical data. The absence of these variables may have influenced the observed tooth-survival outcomes and should be regarded as a significant limitation of this study. In addition, although the Hosmer–Lemeshow test indicated acceptable model fit, some model diagnostics could not be performed due to limitations inherent in the retrospective dataset. This constraint should also be acknowledged when interpreting the regression results.

Nevertheless, long-term follow-up and stratification based on occlusal support provide valuable longitudinal evidence to predict prognosis and plan treatment in partially edentulous patients. The present study highlights that, using multivariable analysis, Eichner classification can be used as an independent predictor of tooth loss, suggesting that patient-specific risk stratification may improve long-term outcomes.

Future research should focus on prospective studies using larger cohorts and adjusting for relevant confounding variables. Further evaluation of prosthetic design, occlusal planning, timing of implant placement, and functional loading will help refine preventive strategies and optimize treatment for high-risk patients.

## 5. Conclusions

Implant treatment demonstrates high durability and functional reliability in partially edentulous patients. Occlusal support is a possible predictor of residual tooth loss, emphasizing the importance of individualized monitoring and preventive interventions. The Eichner classification is a potential clinical tool not only for occlusal assessment but also for long-term prognosis.

## Figures and Tables

**Table 1:** Patient population.

Patient Population	Male	Female	Total
	80 (35.56%)	145 (64.44%)	225

**Table 2:** Eichner classification after the delivery of definitive prostheses.

Eichner Classification After Implant Treatment	A	B1	B2	B3
	159 (70.67%)	48 (21.33%)	16 (7.11%)	2 (0.89%)

**Table 3:** The number of lost teeth in each patient during the observation period.

No. of Lost Teeth	0	1	2	3	4	5	6	7	8	9	10	11	12	13	Total Number of Lost Teeth
Number of patient(s)	55	50	34	22	21	19	5	11	2	3	1	1	0	1	547

**Table 4:** Number of lost implants in each patient during the observation period.

No. of Lost Implants	0	1	2	3	4	5	6	7	8	9	10	11	12	13	Total Number of Lost Implants
Number of patient(s)	203	15	3	3	0	1	0	0	0	0	0	0	0	0	35

**Table 5:** Cross-tabulation table for the relationship between Eichner classification after the definitive prosthesis delivery and tooth loss. Asterisk indicates statistically significant difference.

Tooth Loss (≥3)	Eichner A	Eichner B	Total	*p* Value
No	105	34	130	0.041 *
Yes	54	32	86	
Total	159	66	225	
**Tooth loss (≥2)**	**Eichner A**	**Eichner B**	**Total**	***p* V** **alue**
No	79	26	105	0.159
Yes	80	40	120	
Total	159	66	225	
**Tooth loss (≥1)**	**Eichner A**	**Eichner B**	**Total**	***p* Value**
No	44	11	55	0.080
Yes	115	55	170	
Total	159	66	225	

**Table 6:** Multivariable logistic regression analysis of tooth loss (≥1, ≥2, ≥3). Adjusted for age, sex, and Eichner classification. Asterisk indicates statistically significant difference (* indicates statistical significance *p* < 0.05).

Tooth Loss (≥3)	Variables	OR	95%CI	*p* Value
	Age	1.033	1.001–1.066	0.046 *
	Sex (reference: female)	0.899	0.502–1.609	0.719
	Eichner B (reference: Eichner A)	1.830	1.005–3.332	0.048 *
	C-statistics	0.601		
**Tooth loss (≥2)**	**Variables**	**OR**	**95%CI**	***p* Value**
	Age	1.063	1.029–1.098	<0.001 *
	Sex (reference: female)	1.488	0.833–2.659	0.179
	Eichner B (reference: Eichner A)	1.365	0.742–2.513	0.317
	C-statistics	0.645		
**Tooth loss (≥1)**	**Variables**	**OR**	**95%CI**	***p* Value**
	Age	1.039	1.005–1.074	0.023 *
	Sex (reference: female)	0.879	0.454–1.702	0.702
	Eichner B (reference: Eichner A)	1.884	0.887–4.001	0.099
	C-statistics	0.623		

## Data Availability

De-identified data supporting the findings of this study are available from the corresponding author upon reasonable request.

## Supplementary Materials

The following supporting information can be downloaded at: https://www.mdpi.com/article/10.3390/jcm14248939/s1, Table S1: Risk ratios and risk differences for tooth loss by Eichner classification. Results from binomial regression models for three tooth loss thresholds (≥1, ≥2, and ≥3 teeth), adjusted for age and sex.; Table S2: Incidence rate ratios from negative binomial regression models. Results treating tooth loss counts as continuous variables, with age, sex, and Eichner classification as explanatory variables.
